# Mineral Trioxide Aggregate vs. Calcium Hydroxide in Primary Molar Pulpotomy: A Systematic Review 

**Published:** 2014-03-08

**Authors:** Armin Shirvani, Raheleh Hassanizadeh, Saeed Asgary

**Affiliations:** a * Iranian Center for Endodontic Research, Research Institute of Dental Sciences, Shahid Beheshti University of Medical Sciences, Tehran, Iran; *; b * Knowledge Management Unit, Research Institute for Dental Sciences, Shahid Beheshti University of Medical Sciences, Tehran, Iran*

**Keywords:** Calcium Hydroxide, Meta-Analysis, Mineral Trioxide Aggregate, MTA, Primary Molar, Pulpotomy

## Abstract

**Introduction**: The aim of this quantitative systematic review/meta-analysis was to compare the treatment outcomes of mineral trioxide aggregate (MTA) and calcium hydroxide (CH) in pulpotomy of human primary molars. The focused PICO question was “in case of pulp exposure in vital primary molars, how does MTA pulpotomy compare to CH in terms of clinical/radiographic success?” **Methods and Materials**: We retrieved published randomized clinical trials (RCTs) of at least 6-month duration; our search included articles published up to March 2013 in five following databases: PubMed (Medline), Cochrane database of systematic reviews, Science Citation Index, EMBASE, and Google Scholar. Mantel Haenszel and Inverse Variance-weighted methods were applied by STATA; the relative risk (*RR*) was calculated with 95% confidence intervals (*CI*). **Results:** A total of 282 English articles were collected. Two authors independently screened the articles and five RCTs were selected; data extraction and quality assessment were then carried out. Four RCTs were appropriate for meta-analysis according to their follow-up times by Mantel Haenszel method. Statistically significant difference was found between success rate of MTA compared to CH, with *RR*=0.08 (95% *CI*, 0.02-0.39), *RR*=0.19 (95% *CI*, 0.08-0.46), and *RR*=0.38 (95% *CI*, 0.21-0.68) for 6-, 12-, and 24-month follow-ups, respectively. A significant difference was also observed for all included RCTs after analyses using the Inverse Variance-weighted method (*RR*=0.44; 95% *CI*, 0.27-0.72). **Conclusions:** Systematic review/meta-analysis of included RCTs revealed that for pulpotomy of vital primary molars, MTA has better treatment outcomes compared to CH.

## Introduction

Pulpotomy is defined as surgical removal of the coronal pulp; it is a universally accepted treatment to retain primary teeth with pulp exposure due to trauma or caries, unless tooth saving is deemed impossible. Currently, pulp devitalization, preservation or regeneration are considered as various treatment approaches using numerous materials including formocresol (FC), ferric sulfate, zinc oxide eugenol, calcium hydroxide (CH), mineral trioxide aggregate (MTA), Portland cement and calcium enriched mixture (CEM) cement [1-3]. The procedure of pulp preservation or regeneration is based on the rationale that the remaining pulp is either healthy or if inflamed capable of healing after surgical amputation and dressing with a proper biomaterial [4]. In other words, the main aim of pulpotomy is to retain a functional tooth in the oral cavity by preservation of the radicular pulp until its exfoliation [5]. It has been shown that time of eruption and orientation of permanent teeth may be effected when their corresponding primary teeth have undergone pulpectomy [6].

FC pulpotomy is the common devitalization method for primary teeth in pediatric dentistry. After application of FC, necrosis happens in at least the coronal third of the radicular pulp; chronic inflammation of remaining pulp has also been reported [7]. Regardless of concerns about safety (*i.e.* mutagenicity, carcinogenicity and immune sensitization potential) of FC application for pediatric patients, preservation of vitality and normal state of radicular pulp is of utmost importance to guide a large body of research for alternative safer agents for pulpotomy of primary teeth [8].

An important alternative to FC for primary teeth pulpotomy was CH as is a white, crystalline, highly alkaline, and slightly soluble basic salt, which is able to induce the formation of a hard tissue bridge [9, 10]; it is shown that this bridge may contain some defects under light as well as scanning electron microscopy (SEM) [11, 12]. Internal root resorption as the most frequent side effect is a reason for failure of pulpotomy with CH in primary teeth [9, 13, 14].

MTA as an endodontic filling biomaterial has made a great impact in dental practices all around the world during recent two decades [15, 16]. The major components of MTA and Portland cement are the same except for bismuth oxide [17]. MTA is proposed to be used as a pulpotomy agent in primary and permanent teeth [18, 19]. It is also claimed that MTA is a bio-inductive material that can induce hard tissue formation in direct contact with pulp [20].

Systematic reviews and meta-analysis based on randomized controlled trials (RCTs), are the best reliable sources for suggesting and making decisions in clinical dental practice [21]. In 2003, the only Cochran systematic review regarding vital pulp therapy for primary teeth stated that: “based on the available RCTs, there is no reliable evidence supporting the superiority of one type of treatment for primary molars with their pulps involved”. This gap highlights the need for high quality RCTs, with appropriate unit of randomization and analysis [1]. Two systematic reviews have summarized the published RCTs and concluded that MTA demonstrated significantly better treatment outcomes compared to FC in primary molar pulpotomy [8, 22, 23]. While, the results of comparing MTA and CH by several RCTs have been published but the evidences are diverse and there is no concluding systematic review to provide a comprehensive conclusion. Therefore, the aim of the present systematic review and meta-analysis of randomized clinical trials was to compare the treatment outcomes of MTA and CH in pulpotomy of primary molars based on RCTs.

## Methods and Materials

***PICO question***

In case of pulp exposure in vital primary molars, how does MTA pulpotomy compare to CH in terms of clinical and radiographic success?

***Literature search***

A comprehensive computerized search (from 1967 through June 2013) was conducted using Medline, the Cochrane database of systematic reviews, Science Citation Index (SCI), EMBASE and Google Scholar. Clinical Queries filter of PubMed, facilitated finding controlled clinical trials. RCTs comparing CH and MTA were identified using the following query: ("mineral trioxide aggregate" [Supplementary Concept] OR ("mineral trioxide" Field: Title/Abstract) AND ("Calcium Hydroxide"[Mesh] OR ("Calcium Hydroxide" Field: Title/ abstract)”. From the found results, randomized controlled trials on primary molars were selected.

***Inclusion and exclusion criteria***

The following studies were included in this research: English papers with original data that evaluated pulpotomy treatment of primary human teeth with vital-pulp exposure due to caries or trauma, which included either CH or MTA, follow-up time of at least 6 months, restorable teeth, and evaluation by clinical symptoms and radiographic methods. Exclusion criteria were as follows: lack of randomization and absence of comparison between the treatment groups.

***Data extraction and quality assessment***

Data were directly extracted and verified from the full texts by two reviewers (AS and SA). Disagreements were solved by re-checking the text and discussion. The quality of selected studies was assessed using a series of validity criteria according to the modified van Tulder list [24] (Appendix 1). Selected studies were evaluated based on: *i)* randomization, *ii)* allocation concealment, *iii)* groups similarity at baseline, *iv)* blindness of outcome assessor(s), v) blindness of care provider(s), *vi)* blindness of patients, *vii)* calibration of outcome assessor(s), *viii)* avoidance of co-interventions, *ix)* follow-up periods being adequate, *x)* description of withdrawal and dropout rates, *xi)* the timing of the outcome assessment being comparable in all groups, *xii)* relevant outcomes, *xiii)* adequate sample size, and *xiv)* using of objective outcome measures. To ensure the validity of included articles, two reviewers assessed the abstracts and full texts independently; disagreement was resolved in consensus meetings.

***Summary measures and synthesis of results***

The main outcome for meta-analysis was clinical/radiologic failure. Since the outcome measures were dichotomous, Mantel Haenszel analysis was used to estimate pooled Relative Risk (RR). The results of 6-, 12- and 24-month follow-ups were compared calculating the pooled RR for each recall interval. One study with 56 months of follow-up was excluded from the analyses; however, the final results of that study had been included in a calculation of pooled RR for all 6-month interval observations using the Inverse Variance-weighted method.

Statistical analysis was performed using STATA version 12 software (STATA Corporation, College Station, Texas, USA). The level of statistical significance was set at 0.05. The heterogeneity among studies and estimation of study variance in all observations was assessed using the Q statistic-test.

**Table 1:** Characteristics of the RCTs included in meta-analysis

	**Randomization**	**Baseline characteristics of study and control groups**	**Co-interventions**	**Patient blinding**	**Follow-up** **(month)**	**Lost to** **follow-up/Total**		**Outcome measurement**			**Score based on modified van Tulder list**
								**Objective**	**Calibrated investigators**	**Blinded investigators**	
						**MTA**	**CH**				
**Percinoto ** ***et al.*** ** [9]**	Ok	NM	No	NM	3,6,12	10/55	10/55	Yes	Yes	Yes	9
**Moretti ** ***et al.*** ** [24]**	Ok	NM*	No	NM	3,6,12,18,24	1/15	1/15	Yes	Yes	Yes	11
**Sonmez ** ***et al.*** ** [25]**	Ok	NM	No	NM	6,12,18,24	0/15	10/23	Yes	No	No	9
**Liu ** ***et al.*** ** [13]**	Ok	NM	NM	NM	56	3/20	3/20	Yes	NM	NM	9
**Oliviera ** ***et al.*** ** [26]**	Ok	NM	No	NM	6,12,24	0/15	0/15	Yes	Yes	Yes	12

* *NM:* *Not Mentioned*

**Table 2:** Inclusion/exclusion criteria of five RCTs included in the meta-analysis

	**Participants**	
	**Inclusion criteria**	**Exclusion criteria**
**Percinoto ** ***et al.*** ** (2006) [9] **	Children between 3-8 years	Spontaneous sensitivity, edema, fistula, tooth mobility, periodontal alteration/radiolucency in the region of the furcation or at the apex
**Moretti ** ***et al.*** ** (2007) [24]**	Children between 5-9 years, with no more than two decayed mandibular primary molars with vital pulp and absence of pain history	Pulp degeneration such as excessive bleeding from the root canal, internal root resorption, interradicular/furcal bone destruction; no physiological root resorption of more than one-third, as observed in periapical radiographies; the presence of systemic pathology and any history of allergic reaction to latex, local anesthetics or to the constituents of the test pulp dressing agents
**Sonmez ** ***et al.*** ** (2008) [13]**	Children between 4-9 years, pulp exposure occurred during caries removal, amalgam restoration was possible, and at least two thirds of the root length was present	Pulp degeneration (excessive bleeding, pathological mobility, pathological external root resorption, internal root resorption, interradicular/periapical bone destruction, swelling or sinus tract, history of spontaneous and nocturnal pain, and tenderness to percussion or palpation)
**Liu ** ***et al.*** ** (2011) [13] **	Children between 4-9 years	Spontaneous pain, discomfort at percussion, pathological mobility, swelling, fistula, radiolucency, root resorption, excessive bleeding after pulp amputation, pulp exposure after complete removal of decay
**Oliviera ** ***et al.*** ** (2013) [26] **	Children between 5-9 years, mandibular primary molar with deep caries that compromised the pulp, vital pulp, and the possibility of tooth restoration	Pulp degeneration such as internal root resorption and furcal bone destruction; physiological root resorption of more than one-third, as observed in periapical radiographs; the presence of systemic pathology and history of allergic reaction to latex, local analgesics or to the constituents of the tested pulp capping agents.

## Results

Searches in Medline, the Cochrane database of systematic reviews, SCI, EMBASE and Google Scholar, yielded 282 English published studies. Based on the inclusion/exclusion criteria, 5 RCTs were identified to properly compare CH and MTA for primary molar pulpotomy [9, 13, 25-27]. The data summaries of selected studies are shown in Table 1. Characteristics of participants are summarized in Table 2. Only one study had reported 56-month follow-up results [13]; however, four studies with binary outcomes were comparable according to their follow-up periods and outcome measures. Since the outcome measures were binary, Mantel Haenszel method was employed to estimate pooled RR. Results of included studies are summarized in Table 3.

**Table 3:** Number of success/failure in MTA and CH pulpotomy groups at three follow-up periods; F=Failures, S=Success

**Follow-up**	**6-month**				**12-month**				**24-month**				
**Group**	**CH **		**MTA **		**CH **		**MTA **		**CH **		**MTA **	
**Gender**	**F**	**S**	**F**	**S**	**F**	**S**	**F**	**S**	**F**	**S**	**F**	**S**
**Moretti ** ***et al.*** ** [24]**	6	8	0	14	8	6	1	13	9	5	5	9
**Sonmez ** ***et al.*** ** [25]**	0	13	0	15	4	9	2	13	7	6	5	10
**Percinoto ** ***et al.*** ** [9]**	5	40	0	46	6	39	2	44	-	-	-	-
**Oliviera ** ***et al.*** ** [26]**	7	8	0	15	9	6	0	15	10	5	0	15

**Table 4:** Meta-analysis of all included RCTs (exponential form)

	**Pooled** *** RR*** ** (95% ** ***CI*** **)**			**Asymptotic**		**No. of observations**
**Method**	Est.	Lower	Upper	*Z*-value	*P*-value	
**Fixed**	0.443	0.272	0.720	-3.284	0.001	12
**Random**	0.443	0.272	0.720	-3.284	0.001	

*Test for heterogeneity: Q= 5.226 on 11 degrees of freedom (P= 0.920); Moment-based estimate of between studies variance= 0.000*

**Figure 1:**
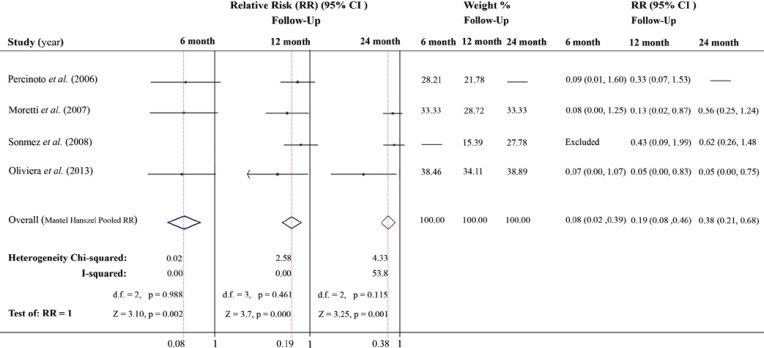
Forest plots: Horizontal line for trials in each follow-up period illustrates the 95% *CI*; shorter line indicating higher precision of the trial. Diamonds are the pooled result, with horizontal tips signifying 95% *CI*, and the vertical tips (superimposed on vertical red section line) indicating pooled *RR*. The vertical line at 1 indicates no treatment outcome difference between the two experimental groups

According to meta-analysis using Mantel-Haenszel method, pooled *RR* for 6, 12 and 24 month follow-ups estimated the Relative Risk as *RR*= 0.077 (*CI* 95%: 0.015- 0.389, *P*=0.002), *RR*= 0.192 (*CI* 95%: 0.080-0.459, *P*= 0.001), and *RR*= 0.376 (*CI* 95%: 0.208-0.678, *P*=0.001), respectively. Forest plots of the results are presented in Figure 1. The results of Pooled *RR* for all of the 6-month interval observations using the Inverse Variance-weighted method including the study by Liu *et al.* [13], is tabulated in Table 4.

In order to include the results reported by Liu *et al.* and to evaluate the effect of follow-up duration on treatment outcome, the random/fixed effect models and the Q-test for heterogeneity were applied via the Inverse Variance-weighted method (including the Liu *et al.*'s study) which revealed a significant difference between the groups. The insignificant difference in the results of the random/fixed effect models as well as Q-test revealed that the duration of follow-up has no impact on treatment outcomes in both groups. 

## Discussion

As the highest ranked evidence, systematic reviews tend to collect, critically apprise and synthesize the results of primary RCTs. In other words, they are valuable methods to address the best current evidence regarding a specific foreground question. Dental clinicians as well as oral health care providers should be aware of the best existing evidence to support their clinical practice. In the new millennium, such studies are usually conducted to investigate diagnostic/prognostic questions, cost-effectiveness, as well as making strategies [28]. In answer to the prognostic PICO question, the present systematic review of the RCTs with meta-analysis revealed that MTA pulpotomy of primary teeth is superior to CH in terms of radiographic treatment outcomes. However, the review search was limited to five RCTs. One trial did not include in the meta-analysis using Mantel Hanszel model, because it did not provide binary outcomes. However, the results of the RCT were included in the calculation of pooled *RR* for all 6-month interval observations from all of the studies (*n*=5), using the Inverse Variance-weighted method; obtained results was constant.

Quality analysis is an appropriate approach to evaluate possible bias [29]; there are various quality scales for measuring the quality of RCTs; however, there is no common consensus on which type of scale to use. The modified van Tulder list, which consists of patient selection, blinding, interventions, and statistics items, was used in this study to appraise the quality of each included RCTs. The van Tulder list, as the latest modification of the Delphi list, is a reliable and valid tool; it is employed by The Cochrane Collaboration Review Groups as well. In this systematic review, included RCTs gained a quality score more than nine; therefore, meta-analysis of high-quality RCTs may produce valid results and conclusions.

Publication bias as the main problem in reporting of RCTs, is increasingly documented as a core complexity in systematic reviews and meta-analysis [30]; it has been defined as the tendency of journals' reviewer/editors to accept RCTs for publication based on the direction/strength of the findings. However, publication bias may be primarily due to the disappointment of researchers to submit negative results of conducted RCTs. Accordingly, the main inference of publication bias is that the conclusions of systematic reviews/meta-analyses based only on published RCTs may be misleading. Our study, however, indicated no evidence of publication bias, as most of included RCTs (3 out of 5) did not report significance in their results.

I-square illustrates the percentage of total variation amongst the studied trials which is attributed to heterogeneity but not chance [31]. A value of 100% indicates absolute heterogeneity and smaller values show decreasing trend. As shown in Figure 1, 0.0% I-square for 6 and 12-month follow-ups indicated complete consistency of included RCTs, however, in case of 24-month follow-up we observed a moderate inconsistency which is equal to 53.8%.

**Appendix 1:** Modified van Tulder List a

	Yes/No/Don’t know
Was an appropriate method of randomization performed?	
Treatment allocation: Was the treatment allocation concealed?	
Were the groups similar at baseline regarding the most important prognostic indicators?	
Was the outcome assessor blinded?	
Was the care provider blinded?	
Were the investigators calibrated?	
Was the patient blinded?	
Was the co-interventions avoided?	
Was the follow-up period adequate?	
Were withdrawal and dropout rates described and acceptable? (>85%) WCA b	
Was the timing of the outcome assessment comparable in all groups?	
Were relevant outcomes used?	
Was the sample size adequate?	
Were the outcome measures objective?	
Did the analysis include an intention-to-treat analysis?	

a : Modified in Knowledge Management Unit (KMU) of Research Institute of Dental Sciences, Shahid Beheshti University of Medical Sciences, Iran;

b *:* WCA: Worse case analysis

## Conclusion

Considering the good quality of the RCTs, homogeneity of the included trials, and lack of publication bias, the results revealed that MTA pulpotomy in human primary teeth presented superior treatment outcomes compared to those treated with CH.
